# Measles Virus Matrix Protein Inhibits Host Cell Transcription

**DOI:** 10.1371/journal.pone.0161360

**Published:** 2016-08-23

**Authors:** Xuelian Yu, Shadi Shahriari, Hong-Mei Li, Reena Ghildyal

**Affiliations:** 1 Section of Epidemiology & Statistics, Department of Public Health, Xinjiang Medical University, 393 XinYi Road, Urumqi, PR China; 2 Department of Biochemistry and Molecular Biology, Monash University, Wellington Parade, Melbourne, VIC 3800, Australia; 3 Respiratory Virology Group, Centre for Research in Therapeutic Solutions, Faculty of ESTeM, University of Canberra, Bruce, ACT 2617, Canberra, Australia; University of Toronto, CANADA

## Abstract

Measles virus (MeV) is a highly contagious virus that still causes annual epidemics in developing countries despite the availability of a safe and effective vaccine. Additionally, importation from endemic countries causes frequent outbreaks in countries where it has been eliminated. The M protein of MeV plays a key role in virus assembly and cytopathogenesis; interestingly, M is localised in nucleus, cytoplasm and membranes of infected cells. We have used transient expression of M in transfected cells and in-cell transcription assays to show that only some MeV M localizes to the nucleus, in addition to cell membranes and the cytoplasm as previously described, and can inhibit cellular transcription via binding to nuclear factors. Additionally, MeV M was able to inhibit in vitro transcription in a dose-dependent manner. Importantly, a proportion of M is also localized to nucleus of MeV infected cells at early times in infection, correlating with inhibition of cellular transcription. Our data show, for the first time, that MeV M may play a role early in infection by inhibiting host cell transcription.

## Introduction

Measles virus (MeV) is a negative sense RNA virus belonging to the morbillivirus genus of *Paramyxoviridae* family and is the causative agent of measles. Despite sustained effort by World Health Organisation (WHO) and its member countries, MeV still causes annual outbreaks in countries where wild type MeV strains circulate [1–3]. Additionally, sporadic outbreaks occur due to importation in countries where endemic MeV has been controlled [3].

The non-segmented negative-sense RNA genome of MeV contains six genes encoding structural proteins, the nucleocapsid (N), phospho- (P), matrix (M), fusion (F), hemagglutinin (H), and large (L) proteins [4]. H and F proteins are surface glycoproteins responsible for virus attachment to and entry into target cells. The genome is encapsidated by the N protein into a nucleocapsid; L and P proteins constitute the viral RNA-dependent RNA polymerase which associates with the nucleocapsid and thereby forms the ribonucleoprotein (RNP) complex [4]. M protein is located in the inner layer of the viral envelope and plays a key role in virus assembly through interactions with viral and cellular factors [5–8]. M protein associates with the inner surface of the plasma membrane [9], and the cytoplasmic tails of the H and F [5, 7]. M protein has also been shown to interact with the RNP complex and regulates viral RNA synthesis via its interaction with the N protein [6]. M protein, through its varied interactions, is thus central to MeV assembly. In addition to P protein, the P gene encodes two nonstructural proteins, V and C via RNA editing and alternative translational initiation in a different reading frame, respectively [10, 11]. The V and C proteins are produced in significant amounts in infected cells, but do not form part of the MeV particle [4], their main function being effective interferon antagonism [12–14].

M also has an important role in MeV cytopathogenicity and changes in the M gene have been linked to subacute sclerosing panencephalitis (SSPE) a long term complication of MeV disease; viruses recovered from patients contain biased hypermutations and premature termination codons in M gene [15] associated with intracellular accumulation of nucleocapsids [9].

M proteins of several cytoplasmic negative sense RNA viruses, including MeV have been shown to localize to the nucleus early in infection, probably to inhibit cellular transcription [16–18]. M protein of vesicular stomatitis virus (VSV) inhibits cellular transcription by sequestering essential transcription factors and by inhibiting mRNA nuclear export [19–21]. The M protein of respiratory syncytial virus (RSV) also localizes to the nucleus of infected cells and probably inhibits cellular transcription [16, 22, 23].

In the current study we show for the first time that a proportion of MeV M localizes to the nucleus when expressed alone and inhibits cellular transcription via binding to chromatin. A similar inhibition of transcription was also observed in infected cells, demonstrating the relevance of our data in the context of infection. Importantly, MeV M was able to inhibit in vitro transcription from a linear DNA template in a dose dependent manner.

## Materials and Methods

### Antibodies, Cells and Viruses

Mouse monoclonal antibody against the MeV M protein was purchased from Novus Biologicals (Littleton, CO). Mouse monoclonal antibody to Lamin B1 was from Santa Cruz Biotechnology. All fluorochrome conjugated secondary antibodies were from Life Technologies.

COS-7 (African green monkey kidney cells, SV40 transformed) and Vero (African green monkey kidney) cells were purchased from Sigma-Aldrich, and maintained in Dulbecco’s modified Eagle’s medium (DMEM; Invitrogen) supplemented with 10% foetal bovine serum (FBS), with 400 μg/ml of penicillin/streptomycin.

MeV (MVi/Zhejiang.CHN/7.05/4; GenBank: DQ211902.1) was isolated from a patient in 2007 in Zhejiang, China and had three amino acid changes in M relative to the vaccine strain Edmonston (G61D, T209A, E210V).

### Plasmids

The M gene was amplified from RNA isocolated from Vero/hSLAM cells infected with MVi/Zhejiang.CHN/7.05/4 and cloned into the Gateway^™^ entry vector, pDONR207 via recombination. The expression plasmid pGFP-MeVM was generated by recombination with the expression plasmid pDESTC-GFP [23]. Plasmid expressing GFP alone (pDESTC-GFP) has been described previously [16]. The bacterial expression plasmid pDEST17-MeVM was generated by recombination of the entry clone with the expression plasmid pDEST17, to express the protein with a hexahis (6xhis) fusion tag.

### Transfection

Overnight subconfluent cultures of COS-7 cells on coverslips in 12-well cluster plates were transfected with pGFP-MeVM using Lipofectamine 2000; DNA and reagent were used at a ratio of 1:1. To determine transfection efficiency and to serve as a control for protein expression and subcellular localization, a set of cells was transfected to express GFP alone [23]. Localisation of GFP and GFP-MeVM was followed by confocal live cell microscopy (CLSM) at 24 h post transfection (p.t.).

### Indirect immunofluorescence assay

Infected or transfected cells were fixed and permeabilised at indicated times using phosphate buffered saline pH 7.2 (PBS) containing 4% formaldehyde and 0.5% Triton X-100. Fixed and permeabilised cells were washed with PBS and incubated with indicated antibodies diluted in PBS, followed by incubation with species specific secondary antibodies conjugated to indicated fluorochromes. Cells were washed and coverslips mounted on slides using the Prolong Gold antifade with DAPI (Life Technologies).

### Western blot analysis

Transfected COS-7 cells were collected for cytoplasmic/nuclear soluble protein extraction at 24 h p.t. Cytoplasmic and nuclear protein extractions were performed using the NE-PER kit as per manufacturer’s recommendation (ThermoFisher). Extracts were heated at 100°C for 5 min in Laemmli buffer (63 mM Tris-HCl, pH 6.8 with 0.1% 2-Mercaptoethanol, 0.0005% Bromophenol blue, 10% Glycerol, 2% SDS) prior to SDS-PAGE on 10% acrylamide gels followed by protein transfer to nitrocellulose membranes in Tris-Glycine-ethanol buffer (25 mM Tris-HCl, 192 mM Glycine, 20% Ethanol) for 90 min at 400 mA. Blots were blocked for 1 h in 4% skim milk (Diploma) in PBS (10 mM Na2HPO4, 1.7 mM KH2PO4, pH 7.2, 2.7 mM KCl, 137 mM NaCl), prior to incubation with primary antibodies diluted in 1% skim milk in PBS-T (PBS containing 0.1% Tween 20) overnight at 4°C with rocking. After washing in PBS-T, blots were incubated with species specific secondary antibodies conjugated to horseradish peroxidase diluted 1:5000 in 1% skim milk in PBS-T, followed by washing and detection of bound antibodies with Enhanced Chemiluminescence (ECL, Perkin Elmer). Protein bands were detected using the Licor OdysseyFc. Digital images were analysed using ImageJ to estimate protein levels relative to tubulin or lamin used as loading controls. Values were corrected for background and normalised to corresponding loading control bands and are expressed as arbitrary units.

### In-cell nuclear association assay

We used a nuclear association assay to examine the nuclear localization of GFP-MeVM [24]. GFP or GFP-MeVM expressing COS-7 cells were fixed with 4% formaldehyde in PBS, without treatment, at 24 h p.t., or permeabilised with buffer A (10 mM Tris-HCl [pH 7.4], 150 mMNaCl, 5 mM MgCl2, 1% NP-40, protease inhibitor) for 15 min and washed with buffer B (buffer A without NP-40) prior to fixing. Some cells were further treated for 1 h with buffer C (buffer B with 40 U DNase I [Roche]/ml) followed by incubation for 10 min with buffer D (10 mM Tris-HCl [pH 7.4], 2 M NaCl, 5 mM MgCl2, protease inhibitor) prior to fixing. Fixed cells were incubated with anti-Lamin B1 antibody followed by goat anti mouse IgG antibody conjugated to Alexa-568. Coverslips were mounted on slides using Prolong Gold antifade with DAPI and imaged by CLSM for localization of GFP-MeVM, Lamin B1 as a marker for nuclear proteins and DAPI as a marker for chromatin. In separate experiments, COS-7 cells treated as above were stained for microfilaments with phalloidin conjugated Alexa-594 and for lamin as above.

### In-cell RNA transcription assay

The Click-iT RNA Imaging Kit (Invitrogen) was used according to the manufacturer's specifications to assess *de novo* RNA synthesis *in situ*. Briefly, GFP/GFP-MeVM expressing COS-7 cells (23 h p.t.) or MeV infected Vero/hSLAM cells (11 h post infection) were treated with 1 mM EU (5-ethynyluridine) for 1 h, fixed with 4% formaldehyde, permeabilised with 0.5% Triton X-100, and stained for *de novo* synthesized RNA using Alexa 594-azide. Cells were then imaged by CLSM. Control cells were treated with actinomycin D (5 μg/ml) for 1 h prior to EU treatment.

### Confocal laser scanning microscopy (CLSM)

Fixed or live (transfected or infected) cells were imaged using a Nikon Ti-E confocal laser scanning microscope (Nikon, Tokyo, Japan) using a 60x oil immersion objective. The ImageJ v1.62 public domain software was used as previously [23–25] to analyze digital images in order to determine the nuclear/cytoplasmic fluorescence ratio (Fn/c), which was calculated by using the equation: Fn/c = (Fn − Fb)/(Fc − Fb), where Fn is the nuclear fluorescence, Fc is the cytoplasmic fluorescence, and Fb is the background fluorescence (autofluorescence). Briefly, the fluorescence in a small defined region of the nucleus is measured (Fn); fluorescence in the cytoplasm is then measured in a region of the same size (Fc), as is the background fluorescence (Fb) [26]. Statistically significant differences were determined with GraphPad Prism. In some cells, the proportion of GFP-MeVM or M in the nucleus relative to that in the whole cell was estimated by the equation: Fn/Ft x 100, where Ft is the total fluorescence in the cell.

### Purification of recombinant 6xhis-MeVM

*E*. *coli* BL21 cells transformed with plasmid pDEST17-MeVM were grown at 37°C in Luria Bertani broth containing 100 μg/ml ampicillin, followed by induction with IPTG (1 mM) for 4 h. The cell pellet was resuspended in lysis buffer (50 mM Tris-HCl [pH 7.4], 500 mM sodium chloride, 25 mM imidazole) supplemented with 2 mg/ml lysozyme, Triton X-100 (0.1% v/v final concentration), 20 μg/ml DNase I, MgCl2 (1 mM final concentration), Roche complete protease inhibitor cocktail. The soluble fraction was isolated by centrifugation at 10,000 x g for 30 min at 4°C, and 6xhis-tagged proteins isolated using Qiagen Ni-NTA spin columns under native conditions following the manufacturer’s instructions. Eluates were dialysed overnight to remove the imidazole and stored in aliquots at -20°C.

### In vitro transcription assay

Various amounts of recombinant purified 6xhis-M protein were incubated with 100 pM of linearized template DNA for 15 min at room temperature and then used in a transcription assay (T7 polymerase, Promega). Reactions (100 μl) were incubated for 1 h at 37°C, and contained transcription buffer, 10 mM DTT, 100 U of Recombinant RNasin Ribonuclease Inhibitor, 0.5 mM rNTP and 40 U of T7 RNA Polymerase. Transcripts were subsequently analysed by electrophoresis in 1% agarose gel containing GelRed imaged in the Bio Rad GelDoc. ImageJ was used to determine the relative band intensities. In separate experiments the transcription products were treated with DNase 1 (Sigma, 0.1 mg/ml) or RNase 1 (Sigma, 0.1 mg/ml) or proteinase k (Invitrogen, 0.2 mg/ml) for 1 h at 37°C prior to gel electrophoresis as above. Digital images shown have been inverted for clarity. Controls included 6xhis-M protein incubated with 100 pM of DNA or 0.1 μg of RNA for 30 min prior to gel electrophoresis.

## Results

### A proportion of MeVM is localised to the nucleus through binding to chromatin

**Fig 1:**
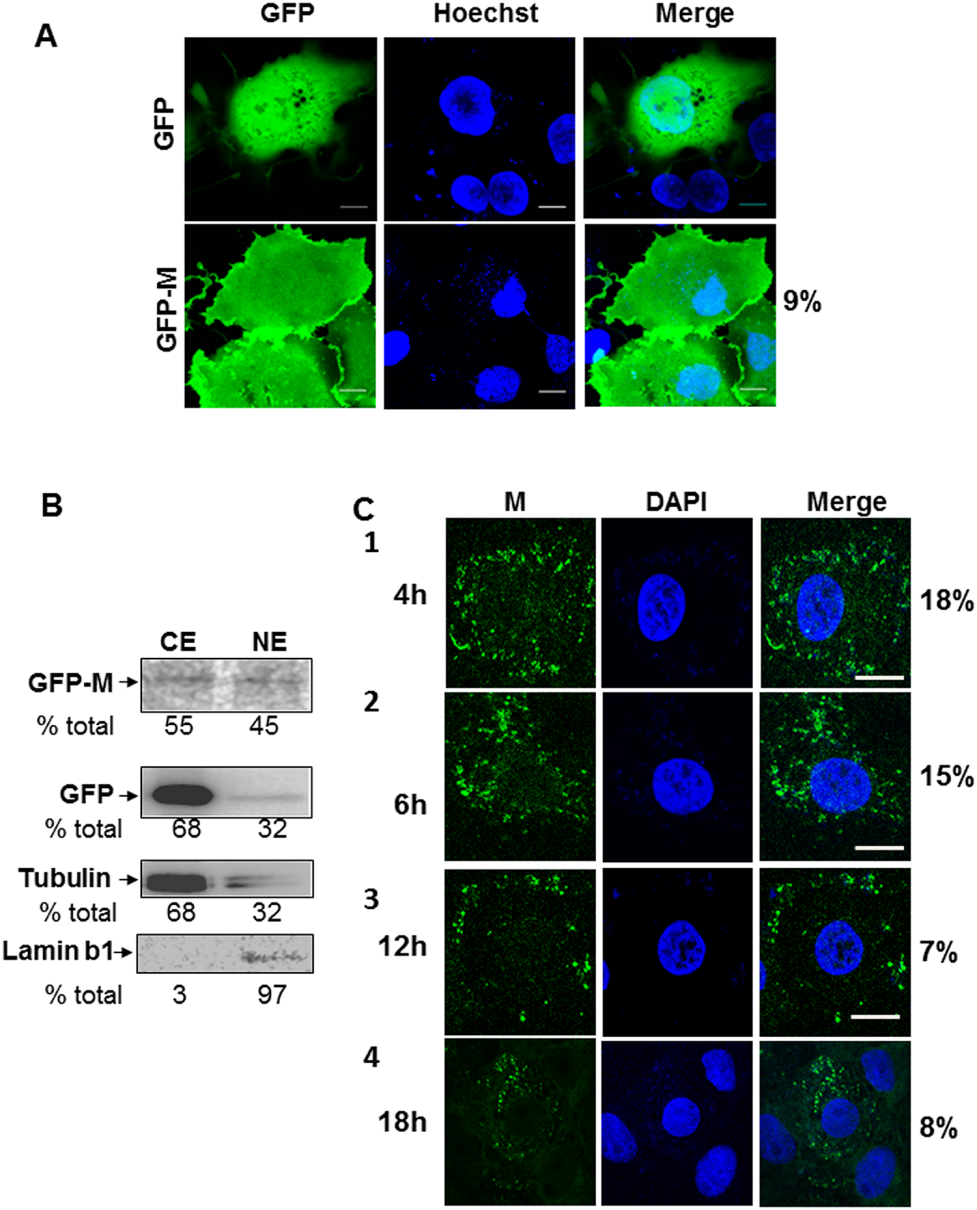
MeV M protein localizes to the nucleus in transfected and infected cells. A. Sub-confluent overnight cultures of COS-7 cells grown on glass coverslips were transfected with pEPI-DESTC-GFP or pEPI-DESTC-GFP-M using Lipofectamine 2000. Transfected cells were incubated with 1 μg/ml Hoechst 33342 for 10 mins prior to confocal microscopy at 24 h p.t. Subcellular localization of GFP, GFP-M and Hoechst 33342 was determined by live cell CLSM. % GFP-MeVM in nucleus is indicated on right of image and represents data from two separate experiments. B. Transfected COS-7 cells as above were collected at 24 h p.t. and cytoplasmic and nuclear fractions extracted using NE-PER, as per the manufacturer’s recommendations. Extracts were heated at 100°C for 5 min in Laemmli buffer prior to SDS-PAGE using 10% acrylamide gels followed by protein transfer to nitrocellulose membranes in Tris-Glycine-ethanol buffer. Blots were blocked for 1 h in 4% skim milk in PBS, prior to incubation with mouse-anti-GFP antibodies (Roche) diluted 1:1000 in 1% skim milk in PBS-T overnight at 4°C with rocking. After washing in PBS-T, blots were incubated with goat-anti-mouse immunoglobulin antibody conjugated to horseradish peroxidase and bands visualised with ECL on Licor Odyssey imager. Bands corresponding to GFP-M, GFP alone and tubulin are shown, with numbers indicating % of the total protein; CE—cytoplasmic extract, NE—nuclear extract. Data are representative of three independent experiments. C. Overnight, subconfluent monolayers of VeroSLAM cells on coverslips were infected with MeV at MOI = 1 and cells fixed and permeabilised at indicated time points (4 h–18 h, rows 1–4). Cells were probed with monoclonal antibody to MeV M followed by Alexa-488 conjugated secondary antibody and mounted on slides with ProLong Gold Antifade with DAPI. Cells were imaged with CLSM for M (green) and DAPI (blue); a merged image was generated using ImageJ and is shown on the right. Numbers on the right indicate mean % M in nucleus from two separate experiments. Scale bar = 5 μm.

When expressed in COS-7 cells, GFP-MeVM localized predominantly to the plasma membrane and diffused throughout the cytoplasm, as expected [17]. A small proportion of GFP-MeVM also localized to the nucleus as defined by co-staining with Hoechst 33342, with 9% of total M being nuclear (Fig 1A). GFP alone, as expected, was diffused throughout the cell with no specific plasma membrane localization evident (Fig 1A). Expression and localisation of GFP-MeVM was confirmed by western blotting of subcellular fractions (Fig 1A). Western blot analysis of nuclear and cytoplasmic extracts from cells expressing GFP-MeVM consistent with the CLSM data, with 45% of intracellular M being present in the nucleus (Fig 1B); as compared to GFP alone and tubulin which were mostly cytoplasmic (32% nuclear). To determine if the nuclear localization of GFP-MeVM was indicative of the subcellular localization of M in infected cells, we probed the localization of M in MeV infected cells at various times post infection up to 18 h p.i. followed by CLSM (Fig 1C). M was primarily localized at cytoplasmic inclusions at all time points studied, with a minor proportion of M also localized to the nucleus (numbers on the right of the images). 18% of M was localized to the nucleus at 4 h p.i., decreasing to 5–7% by 18 h. This is consistent with the amount of nuclear M observed in our transfected cell system (Fig 1A) and in a recent publication examining the nuclear transport of paramyxovirus M proteins [18].

**Fig 2:**
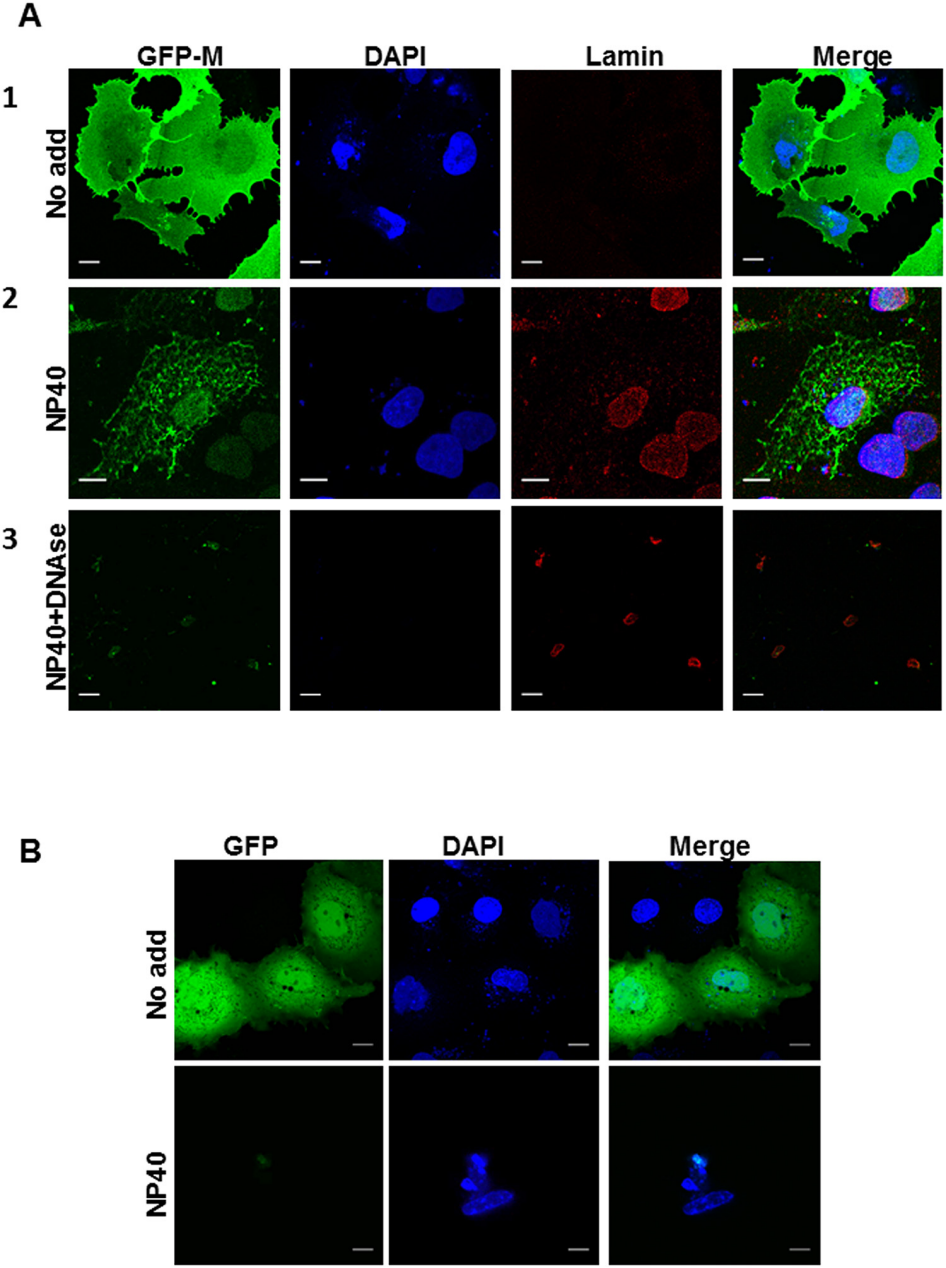
MeVM localizes to the nucleus by associating with chromatin in transfected cells. A. Sub-confluent overnight cultures of COS-7 cells grown on glass coverslips were transfected with pEPI-DESTC-GFP-M as in Fig 1 and fixed at 24 h p.t., (row 1, labeled “no add”), permeabilised with NP-40 (row 2, labelled “NP40”), and treated with DNAse 1 (row 3, labelled “NP40+DNAse 1”). Cells were probed with mouse anti-Lamin B1 antibody, followed by Alexa-568 conjugated secondary antibody and coverslips mounted on glass slides using ProLong Gold Antifade with DAPI. Green (GFP-MeVM), blue (DAPI) and red (Lamin) fluorescence was analysed by CLSM; images were processed with ImageJ to generate the merged images shown in the far right column. Scale bar = 5 μm. B. Sub-confluent overnight cultures of COS-7 cells grown on glass coverslips were transfected with pEPI-DESTC-GFP and fixed at 24 h p.t., (row 1, labeled “no add”) and permeabilised with NP-40 (row 2, labelled “NP40”), Coverslips were mounted on glass slides using ProLong Gold Antifade with DAPI. Green (GFP), and blue (DAPI) fluorescence was analysed by CLSM; images were processed with ImageJ to generate the merged images shown in the far right column. Scale bar = 5 μm.

The observed localization of MeVM in the nucleus could be due to association with nuclear components following nuclear import or due to shuttling across the nuclear envelope. We used an in-cell nuclear association assay to determine the mechanism of MeVM nuclear localization. Cells transfected to express GFP or GFP-MeVM were treated sequentially to remove cytoplasm and DNA and fixed; at each step, localization of GFP-MeVM, GFP (cytosol), phalloidin-A94 (microfilaments), DAPI (chromatin) and Lamin B1 (nuclear matrix/protein) was followed by CLSM. In non-permeabilised cells (Fig 2A, row 1), GFP-MeVM was localized to plasma membrane and in the cytoplasm, with some localized to the nucleus, as expected; Lamin B1 could not be detected due to lack of permeabilisation of the nuclear envelope; clear DAPI staining showed that the nuclei were intact. Permeabilisation with NP40 resulted in loss of cytosol and most of the cytoplasmic GFP-MeVM (row 2). Interestingly, some GFP-MeVM was retained at cytoplasmic structures. Whether these were membranous or cytoskeletal in nature was not examined. In cells expressing GFP alone (Fig 2B), NP40 treatment resulted in complete loss of GFP fluorescence as expected [24]. As expected, phalloidin stained microfilaments were observed under all treatment conditions (S1 Fig). Importantly, GFP-MeVM was clearly retained in the nucleus (2A, row 2, compare the GFP-M and DAPI images). Lamin B1 localization together with the DAPI staining showed that nuclear structure and chromatin were intact. Additional treatment with DNAse 1 resulted in loss of chromatin (2A, row 3) as evidenced by loss of DAPI staining. GFP-MeVM was lost from the nucleus, with only limited GFP flurorescence being observed. Labelling for Lamin B1 showed that nuclear structural proteins were still present; however, there was obvious nuclear shrinkage (compare lamin labelled nuclei in row 2 and 3). Reduction in nuclear radius up to 20% is often observed after treatment with 2 M NaCl [27]. The localisation of GFP-MeVM at cytoplasmic structures observed after NP40 treatment was almost completely lost after DNAse 1 and NaCl treatment. Paramyxovirus matrix proteins are known to form oligomers that dissociate in high ionic conditions and our observation may suggest that MeVM oligomers are involved in interactions with cytoplasmic structures. Together, these experiments suggest that a population of M protein is localised to the nucleus of MeV infected cells where it is probably retained via binding to chromatin with the potential to regulate cellular transcription.

### Nuclear MeVM inhibits cellular transcription

**Fig 3:**
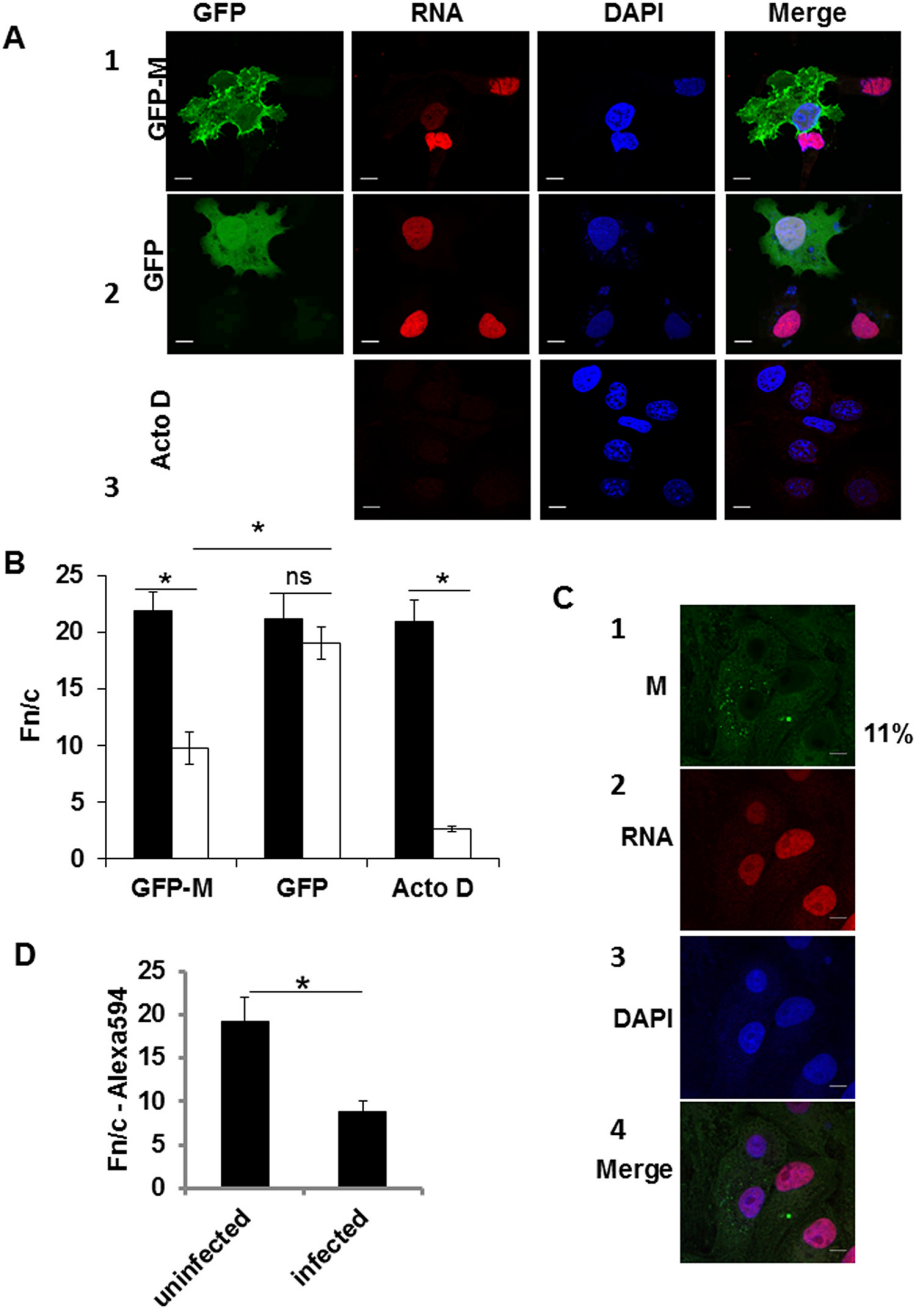
Nuclear MeV M protein inhibits host transcription. A. Transfected cells as in Fig 1 (row 1 GFP-M and row 2 GFP), and mock transfected cells treated with Actinomycin D (row 3, 5 μg/ml, at 22 h p.t.) were incubated in the presence of 1 mM EU for 1 h at 23 h p.t. followed by fixation at 24 h p.t. Cells were probed for EU-labelled RNA using Alexa-594-azide and mounted on glass slides with ProLong Gold Antifade with DAPI, followed by CLSM as in Fig 1. Images are shown for green GFP fluorescence, red fluorescence for Alexa-594 and blue fluorescence for DAPI, with a computer generated merged image shown on the far right. Scale bar = 5 μm. B. Digital images such as those shown in A were analyzed by ImageJ for the relative intensity of Alexa-594 compared to that in the cytoplasm (Fn/c) as an indicator of transcription activity. Data are presented as mean ± SEM of cells positive for GFP/GFP-MeVM or ActoD treatment (white columns) and cells not expressing GFP/GFP-MeVM or not treated (black columns); data are representative of three separate experiments and each datapoint represents n>50 cells. *—p<0.05, ns—not significant. C. MeV infected cells were treated with EU as in A above, fixed and probed for EU-labeled RNA (as in B, row 2) and localization of M examined as in Fig 1B. D. Digital images such as those shown were analyzed for level of transcription using Fn/c for Alexa-594 as a surrogate marker as in B above and is shown in the histogram. Data are representative of two independent experiments; *—p<0.05.

We used an in-cell transcription assay to assess the ability of M to modulate cellular transcription. Cells transfected to express either GFP-MeVM or GFP alone (as above) were analysed for *de novo* mRNA using the Click-iT assay wherein cells are cultured for 1 h in the presence of a uridine analogue EU, that can be detected with fluorophore conjugated azide. EU incorporation (red labelling) was observed exclusively in the nucleus (Fig 3A, images labelled RNA), but did not overlap exactly with the blue staining by DAPI; this is not surprising as DAPI stains double stranded DNA while EU is incorporated into newly forming RNA that would lie close to but not necessarily colocalise with DNA. This becomes clearer in a zoomed-in image of the nucleus (S2 Fig); nucleoli are strongly labelled with EU, but not by DAPI, and areas of dense chromatin are labelled strongly by DAPI but don’t incorporate EU. Incorporation of EU in mRNA was inhibited in cells expressing GFP-MeVM (Fig 3A; compare red staining for mRNA in cell expressing GFP-MeVM and that in an adjacent cell not expressing GFP-MeVM in row 1). This inhibition was not due to transfection or expression of GFP, as cells expressing GFP alone (3A, row 2) showed transcription activity comparable to adjacent non-transfected cells. As expected, treatment with actinomycin D (ActoD) resulted in almost complete inhibition of nuclear transcription (3A, row 3).

There was a significant decrease in Fn/c for EU-labelled mRNA in GFP-MeVM expressing cells (compared to non-expressing cells, Fig 3B), and in cells treated with ActoD (compared to non-treated cells) but not in cells expressing GFP only (compared to non-expressing cells); the EU-labelled mRNA level in GFP-MeVM expressing cells was also significantly different from EU-labelled mRNA level in cells expressing GFP alone. Fn/c, as used here, is a surrogate for transcription activity within a defined period, as more transcription would result in higher intensity of EU labelling in the nucleus and hence, a greater Fn/c value.

A similar inhibition of cellular transcription was observed in MeV infected cells (Fig 3C). Localization of M and EU-labeled mRNA was examined at 12 h p.i. As expected, cytoplasmic M was localized primarily to cytoplasmic inclusions, with a small proportion in the nucleus (Fig 3C, row 1, mean nuclear M = 11%); transcription was significantly inhibited in infected cells compared to uninfected cells on the same slide (Fig 3D, histogram).

### MeV-M is capable of inhibiting in vitro transcription

**Fig 4:**
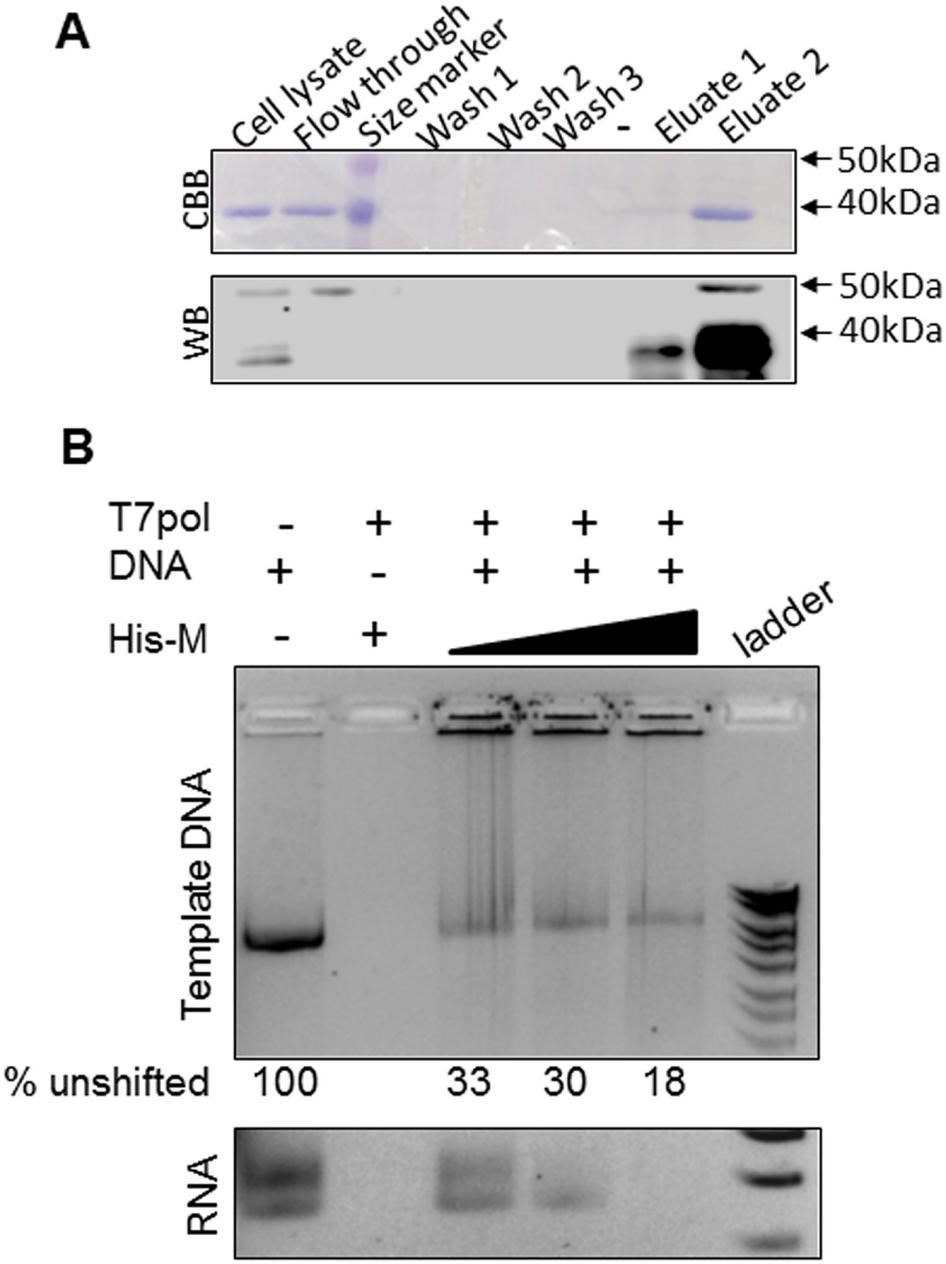
MeV M protein inhibits in vitro transcription. A. His-tagged MeV-M was purified from bacterial lysates as described in methods, using Qiagen Ni-NTA spin columns. Samples from different stages of the purification were analysed by SDS-PAGE and stained with Commassie Brilliant Blue (panel labelled CBB). Samples were also analysed by western blot using antibody specific for the 6xhis fusion tag (panel labelled WB). Numbers on the right indicate molecular weight markers (kDa). B. Different amounts of purified 6xhis-MeV-M (10, 20 or 40 μl) were incubated with a linearised DNA template for 15 mins followed by transcription reaction for 1 h as described in methods. Control sample had no 6xhis-MeV-M or no DNA. Samples were analysed by agarose gel electrophoresis. The upper panel shows the higher molecular weight section of the gel, with numbers underneath indicating % unshifted DNA template band. The lower panel shows RNA products in the lower molecular weight section of the gel. T7pol—T7 polymerase, His-M– 6xhis-MeV-M, ladder—Bioline Hyperladder I.

We next assessed the ability of MeV-M to inhibit in vitro RNA transcription from an unrelated template (Novagen vector pET-30a). Recombinant purified 6xhis-M (Fig 4A, Coommassie Brilliant Blue stained gel and western blot with anti-6xhis antibody show purification of the protein) was added in different amounts to transcription assay and the products analysed by electrophoresis (Fig 4B). Without 6xhis-M, clear RNA products were observed (lane 1); these products decreased in a dose dependent manner with addition of 6xhis-M (lanes 3–5). The higher molecular weight band corresponds to the template DNA and was lost on DNase 1 treatment (S3B Fig). The lower molecular weight bands are RNA and were lost on RNase 1 treatment. Interestingly, the template DNA band was shifted to higher molecular weights in the presence of 6xhis-M (compare the DNA band in lane 1 with those in lanes 3–5). In order to determine whether this shift was due to a direct binding of 6xhis-M to the DNA template and thus, the underlying mechanism of inhibition of transcription, we incubated the template DNA with increasing amounts of 6xhis-M and analysed the products by electrophoresis (S3A Fig). No shift of the template DNA was observed, suggesting that 6xhis-M does not bind to naked DNA. 6xhis-M did however, bind to RNA in a dose dependent manner (S3B Fig). Additionally, treatment of transcription products with proteinase k to degrade 6xhis-M had no effect on the banding pattern in gel electrophoresis; our data suggest that 6xhis-M associates with RNA, probably in actively transcribing polymerase complexes. Importantly, neither DNase1 nor proteinase k had any effect on the pattern of RNA bands, suggesting that the decrease in RNA band intensity was not due to shifting of RNA-6xhis-M complexes to higher molecular weight.

Our data suggest that the nuclear localization of MeVM and binding to nuclear factors results in inhibition of cellular transcription in transfected cells expressing GFP-MeVM and in MeV infected cells. The inhibition of transcription is probably due to MeV-M binding to RNA polymerase complexes on chromatin, as evidenced by in vitro transcription assays.

## Discussion

This study is the first to show that MeV M protein has an inherent ability to localize to the nucleus and inhibit host cell transcription. Although M proteins of other paramyxoviruses have been shown to localize to the nucleus of infected cells (Sendai virus, Newcastle disease virus, Nipah virus) [28–31], this is the first report of a specific nuclear function for a paramyxovirus M protein.

Our data show that M protein is present at the cell membrane, associated with intracellular membranes, bound to cytoplasmic contents as well as in the nucleus, when expressed alone. Similar to our observations regarding MeV-M, only a small proportion of matrix proteins of several paramyxoviruses localise to the nucleus [18]; the reason for this differential subcellular distribution or its importance in virus infection is not known. MeV M is a multifunctional protein with a central role in modulation of nucleocapsid function and virus assembly and has previously been shown to localize to membranes and to cytoplasmic inclusions in infected cells. Interestingly, loss of M protein interaction with viral RNPs in the cytoplasm, as is observed in M protein cloned from SSPE patients [9, 32], resulted in the re-localization of M to the nucleus [17]. Mutations within the M gene are common in SSPE patient samples that are typically unable to produce progeny virus particles, supporting an essential role for M protein in MeV assembly and budding [33]. This is further supported by the finding that an M-less recombinant MeV is highly fusogenic and produces significantly lower infectious titres [34]. Clearly, M is essential for production of infectious virus particles; however, it is dispensable for cell to cell spread via fusion. In a study of vaccine, wild type and SSPE derived M proteins; Jiang et al showed that SSPE related mutations resulted in localization of M protein to the nucleus of transfected cells with reduced plasma membrane localization. Taken together with our data, this suggests that M protein has the ability to localize to the nucleus, but is mostly retained at the RNPs and plasma membrane via specific interactions with other viral and cellular proteins in infected cells. The nuclear import mechanism is not known; MeVM amino acid sequence codes for a canonical nuclear import signal (^225^KKRK^228^) and it is possible that it uses an active import pathway. Interestingly, we did not observe plasma membrane localisation of M protein in MeV infected cells; this may be due to the fixation protocol which was optimised for the antibody used.

Using in-cell, CLSM dependent sequential localization and transcription assays, we show that MeV M protein can inhibit global cellular transcription by binding to the chromatin fraction of the nucleus. The association of M with chromatin may be indirect, through interaction with cellular transcription factors as has been shown for other viral proteins [19, 20, 35–37] or direct, through association with DNA, as shown for transcription repressors [38–41]. Recombinant purified MeV-M was able to inhibit in vitro transcription of a linearised DNA template by T7 polymerase in a dose dependent fashion probably via binding to the RNA in an active polymerase complex as it did not bind to DNA alone, but was able to bind RNA directly and in a non-specific manner. Importantly, treatment of the transcription products had no effect on the intensity or pattern of the RNA bands. If the reduction in RNA band intensity in presence of 6xhis-M was a result of the RNA-6xhis-M complex being shifted to higher molecular weight, we would expect to observe comparable RNA products in all lanes. Thus, MeVM is not a transcription repressor but probably functions to transiently inhibit host cellular transcription. However the exact mechanism, including M’s binding partners and essential motifs remain to be elucidated.

Many viruses have developed effective mechanisms to inhibit the potent antiviral responses mounted by virus-infected cells including inhibition of nuclear functions, e.g. disruption of nuclear transport and inhibition of cellular transcription. The role of the M protein of VSV, the prototypic Rhabdovirus, has been studied extensively in this context. VSV M protein causes global inhibition of transcription by all three host RNA polymerases and inhibits nuclear export of RNA [19–21] in transfected cells in the absence of other viral components. One mechanism by which VSV M mediates its diverse functions is through interaction with multifunctional host proteins, such as Rae1, which forms a chromatin associated complex with Nucleoporin 98 [20, 42].

Transcription repressors bind directly to DNA which is often followed by recruitment of large complexes that contain other transcription factors and epigenetic enzymes resulting in repression of the gene through direct hindrance to polymerase movement on DNA, or indirectly through modification of the DNA as seen in KRAB-ZFPs, Sp3 and CtBP1 [39, 40].

Together, our data suggest that MeV M protein has the inherent ability to localize to the nucleus, where it associates with the polymerase machinery and inhibits global cellular transcription. Although not directly tested in this study, our data from the in-cell and in vitro transcription assays suggest a global rather than a gene-specific inhibition of cellular transcription. Future studies in the group are focussed on elucidation of the precise interactions between MeVM and the transcription complexes.

## Supporting Information

S1 Fig Sequential cell permeabilisation assay. COS-7 cells were either fixed with formaldehyde (row labelled no add), permeabilised with NP40 before fixing (row labelled NP40) or permeabilised with NP40, treated with DNase 1, washed with 2M NaCl before fixing (row labelled NP40+DNase). Cells were probed for lamin b1 (mouse anti-lamin b1 antibody) followed by incubation with phalloidin-Alexa594 Alexa-488 conjugated anti-mouse secondary antibody and coverslips mounted on glass slides using ProLong Gold Antifade with DAPI.
(TIF)

S2 Fig Overlap of DAPI staining with EU incorporation in nuclei. COS-7 cells grown on glass coverslips were incubated with EU for 1 h prior to fixation and labelling of the incorporated EU with Alexa-594 conjugated azide and mounting on glass slides using ProLong Gold Antifade with DAPI. Two examples of the resultant nuclear staining are shown, scale bar = 5 μm.
(TIF)

S3 Fig A. Purified 6xhis-MeV-M was incubated with linearised DNA as in Fig 4B, or yeast RNA (Sigma) for 30 min, followed by agarose gel electrophoresis. B. Transcription reaction products from Fig 4B were treated with DNase 1, Proteinase k or RNase 1 for 1 h at 37°C prior to analysis by agarose gel electrophoresis.
(TIF)
