# Supplementary figures and images for: Measles Virus Matrix Protein Inhibits Host Cell Transcription
Source: PLoS One. 2016 Aug 23;11(8):e0161360. doi: 10.1371/journal.pone.0161360 (PMC4994966; doi:10.1371/journal.pone.0161360)

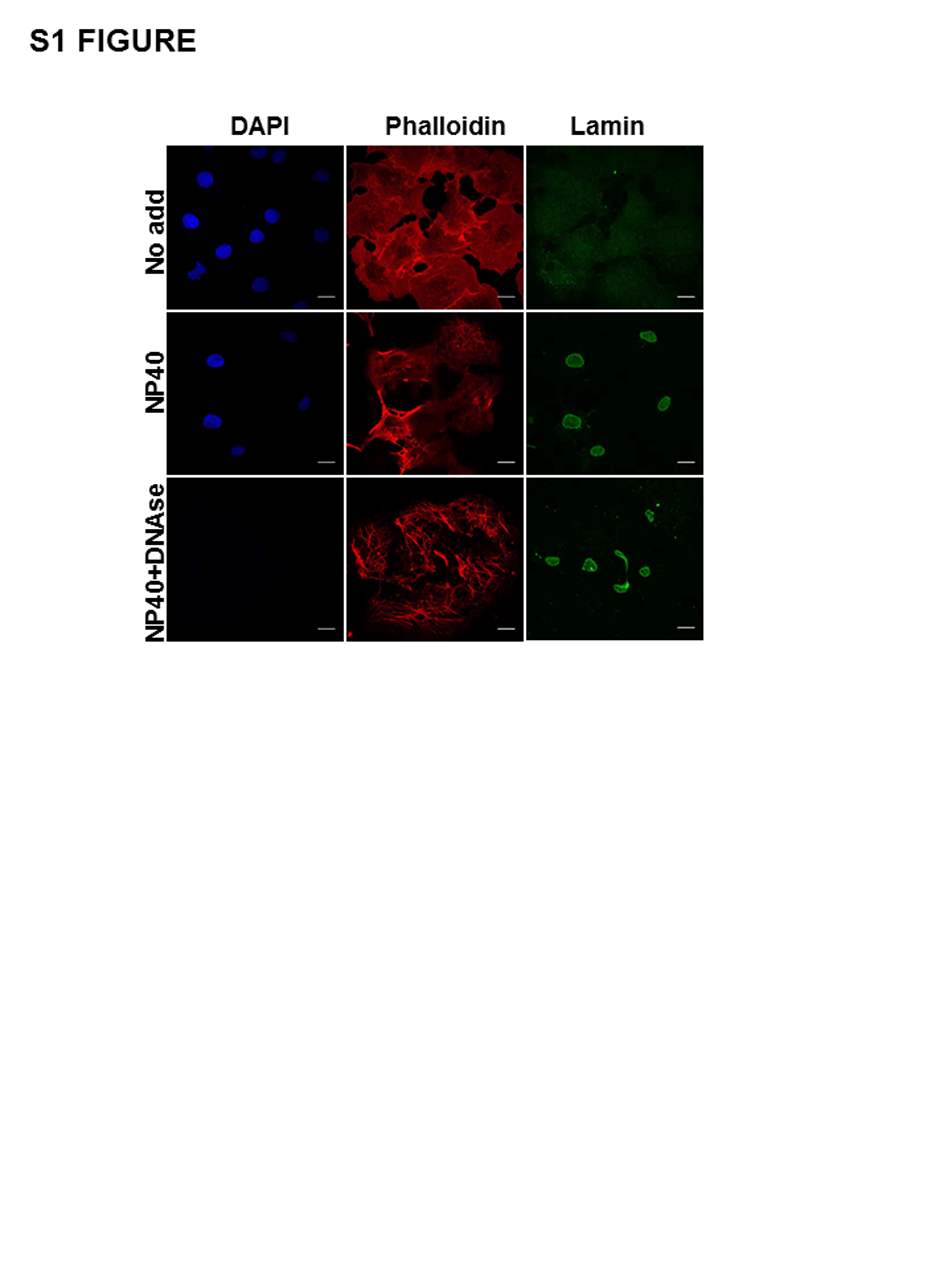

Supplement: S1 Fig — COS-7 cells were either fixed with formaldehyde (row labelled no add), permeabilised with NP40 before fixing (row labelled NP40) or permeabilised with NP40, treated with DNase 1, washed with 2M NaCl before fixing (row labelled NP40+DNase). Cells were probed for lamin b1 (mouse anti-lamin b1 antibody) followed by incubation with phalloidin-Alexa594 Alexa-488 conjugated anti-mouse secondary antibody and coverslips mounted on glass slides using ProLong Gold Antifade with DAPI. (TIF) [file pone.0161360.s001.tif]

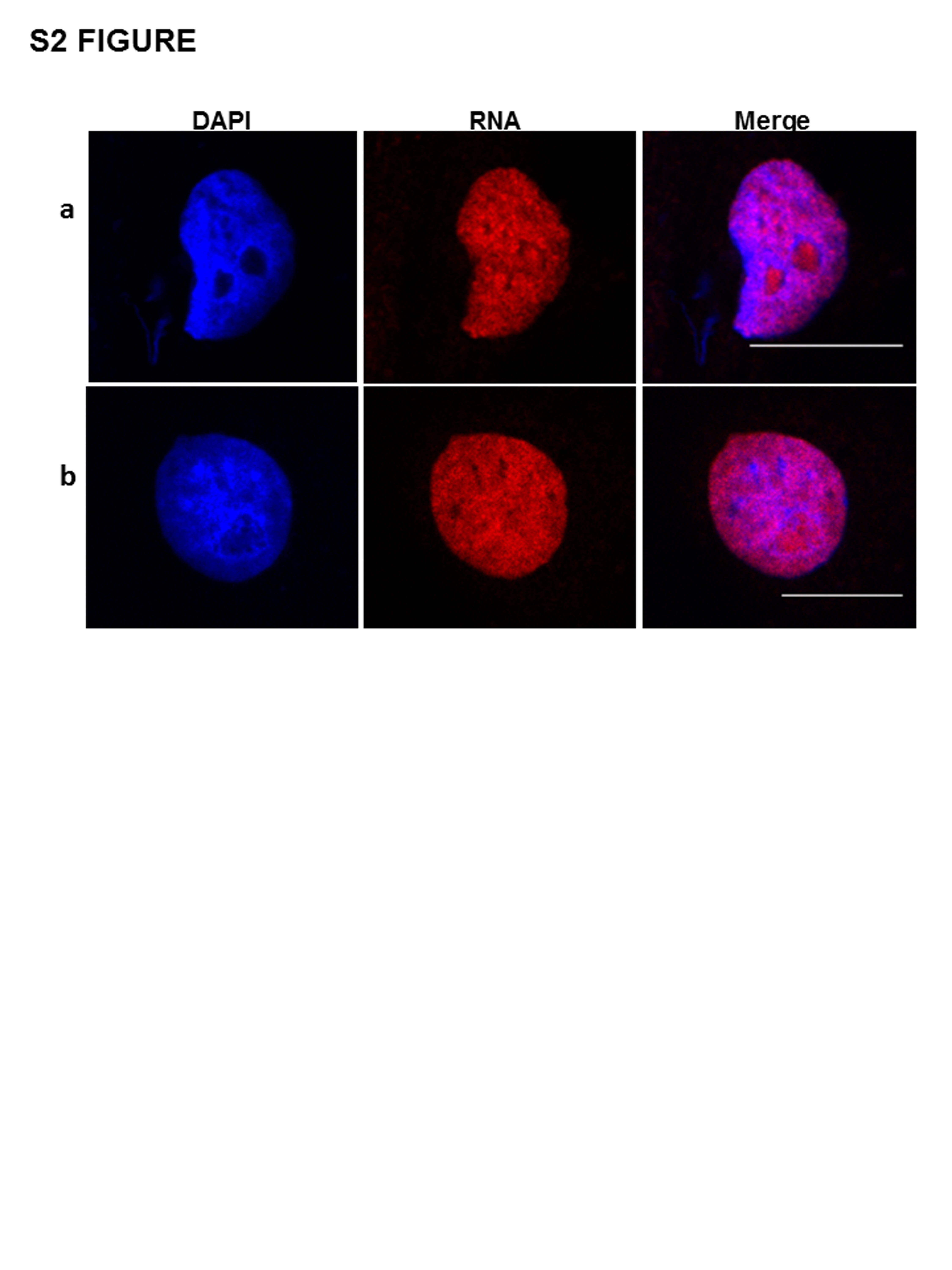

Supplement: S2 Fig — COS-7 cells grown on glass coverslips were incubated with EU for 1 h prior to fixation and labelling of the incorporated EU with Alexa-594 conjugated azide and mounting on glass slides using ProLong Gold Antifade with DAPI. Two examples of the resultant nuclear staining are shown, scale bar = 5 μm. (TIF) [file pone.0161360.s002.tif]

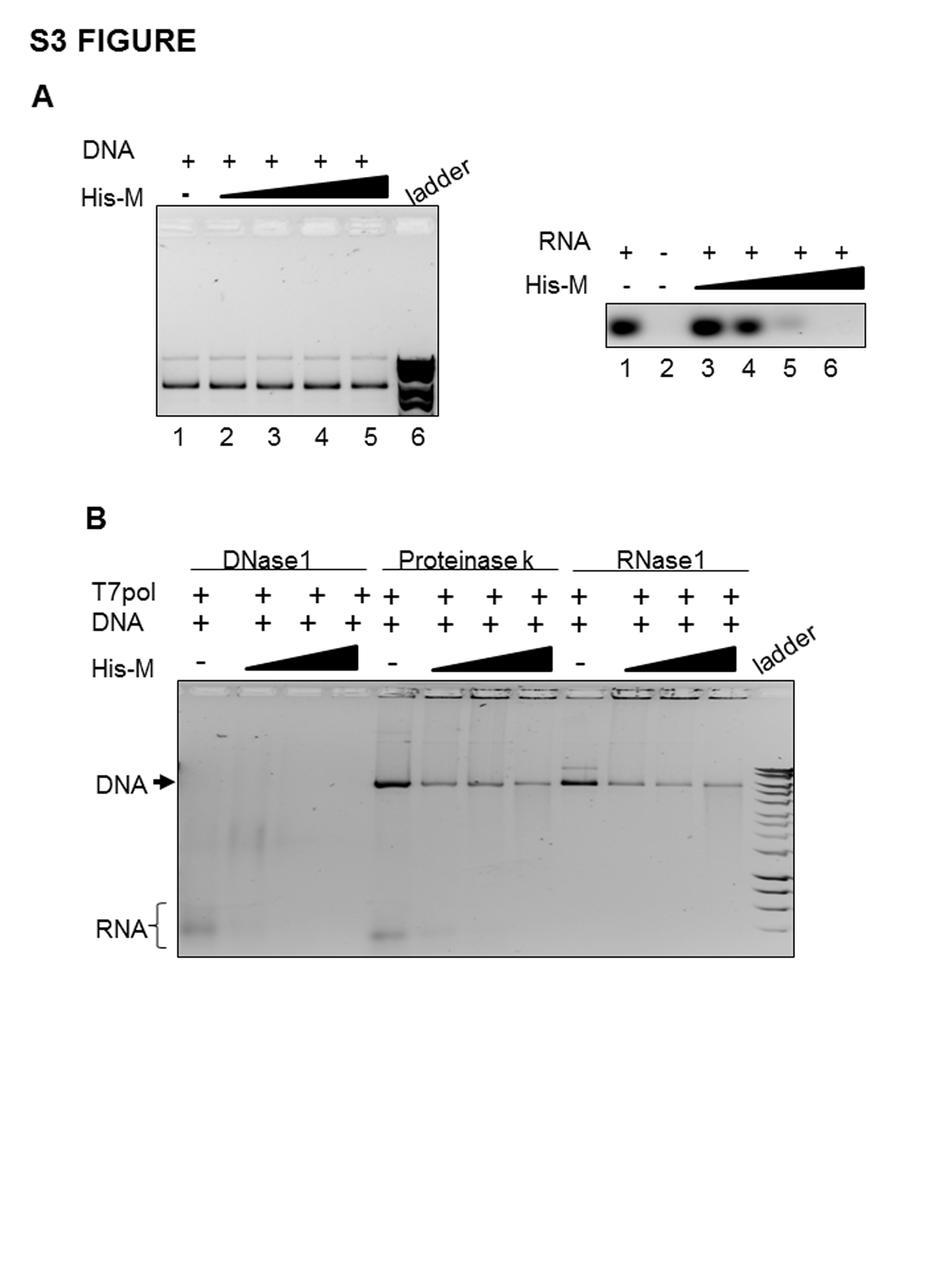

Supplement: S3 Fig — A. Purified 6xhis-MeV-M was incubated with linearised DNA as in Fig 4B, or yeast RNA (Sigma) for 30 min, followed by agarose gel electrophoresis. B. Transcription reaction products from Fig 4B were treated with DNase 1, Proteinase k or RNase 1 for 1 h at 37°C prior to analysis by agarose gel electrophoresis. (TIF) [file pone.0161360.s003.tif]
